# Ultra-Thin Ion Exchange Membranes by Low Ionomer Blending for Energy Harvesting

**DOI:** 10.3390/nano14050478

**Published:** 2024-03-06

**Authors:** Jaehoon Jung, Soyeong Choi, Ilsuk Kang, Kiwoon Choi

**Affiliations:** 1 NextE&M Research Institute, Environmental Industry Research Complex, 410 Jeongseojin-ro, Seo-gu, Incheon 22689, Republic of Korea; 2 National Nanofab Center, Korea Advanced Institute of Science and Technology, 291 Daehak-ro, Yuseong-gu, Daejeon 34141, Republic of Korea

**Keywords:** ion exchange membranes, salinity gradient energy, PVDF-Nafion blends, thin film, low ionomer content, permselectivity, energy efficiency

## Abstract

Exploring the utilization of ion exchange membranes (IEMs) in salinity gradient energy harvesting, a technique that capitalizes on the salinity difference between seawater and freshwater to generate electricity, this study focuses on optimizing PVDF to Nafion ratios to create ultra-thin membranes. Specifically, our investigation aligns with applications such as reverse electrodialysis (RED), where IEMs facilitate selective ion transport across salinity gradients. We demonstrate that membranes with reduced Nafion content, particularly the 50:50 PVDF:Nafion blend, retain high permselectivity comparable to those with higher Nafion content. This challenges traditional understandings of membrane design, highlighting a balance between thinness and durability for energy efficiency. Voltage–current analyses reveal that, despite lower conductivity, the 50:50 blend shows superior short-circuit current density under salinity gradient conditions. This is attributed to effective ion diffusion facilitated by the blend’s unique microstructure. These findings suggest that blended membranes are not only cost-effective but also exhibit enhanced performance for energy harvesting, making them promising candidates for sustainable energy solutions. Furthermore, these findings will pave the way for advances in membrane technology, offering new insights into the design and application of ion exchange membranes in renewable energy.

## 1. Introduction

In the quest for sustainable electricity production, the field of renewable energy has increasingly explored innovative methods. One such promising avenue is salinity gradient energy harvesting, a technique that leverages the difference in salt concentration between freshwater and seawater. The efficiency of this technology critically hinges on the properties of ion exchange membranes (IEMs), particularly the need for reduced electrical resistance to optimize energy conversion. Simultaneously, there is a pressing challenge to achieve a balance between creating thinner membranes, which can further reduce resistance, and maintaining their mechanical durability to enhance operational feasibility [1,2,3]. Conventionally, the development of IEMs has utilized blending techniques to improve the properties of thin membranes, frequently relying on a substantial content of ionomers [4,5,6]. This standard practice is based on the belief that high ionomer levels are necessary for achieving the required ion exchange capacity (IEC) and selectivity of the membranes [7,8,9]. However, this conservative methodology often overlooks the potential benefits of reducing ionomer content due to concerns about creating non-homogeneous structures that may compromise membrane integrity and functionality [10]. Despite the potential benefits associated with reducing film thickness through reduced Nafion content, we still have a general concern that such modifications may result in significant reductions in conductivity, canceling out the benefits gained from thickness reduction. Thus, it becomes imperative to ascertain the limitations of the benefits derived from thickness adjustments [11].

Recognizing this, our study challenges these traditional design constraints with an innovative approach. We propose using materials with established mechanical robustness at ultra-thin dimensions as the primary constituents, with ionomers added only in amounts that exceed the threshold required to facilitate essential IEM characteristics. This novel approach posits that a minimal yet continuous ionomer phase can support efficient ion transport, thus enabling both high ion selectivity and satisfactory proton conductivity, even with a reduced ion exchange capacity (IEC). Furthermore, our research demonstrates that the mechanical and conductive properties of IEMs are not solely dependent on ionomer content but can also be enhanced by the physical configuration of ultra-thin films. By carefully fabricating blending films with thicknesses below two microns and adjusting the composition ratio of Nafion and polyvinylidene fluoride (PVDF), we have shown that it is possible to achieve applicable conductivity and ion selectivity. This was evidenced by comparing the performance of our membranes with the commercial Nafion membrane NR211 in salinity gradient energy harvesting applications, suggesting that thin-film technology can play a crucial role in advancing IEMs.

## 2. Materials and Methods

### 2.1. Membrane Synthesis

The membranes were prepared from a blend solution comprising PVDF and Nafion. The PVDF used had an average molecular weight (Mw) of approximately 180,000 and a number average molecular weight (Mn) of approximately 71,000 and was sourced from Sigma-Aldrich, St. Louis, MO, USA. It was dissolved in NMP (N-Methyl-2-pyrrolidinone, ACS reagent, ≥99.0%, Sigma-Aldrich) to form a 25 wt% solution. Nafion was procured as D2021 (Nafion™ PFSA Polymer Dispersions, The Chemours Company, Wilmington, DE, USA) and was dried and subsequently dissolved in NMP, resulting in a 25 wt% solution. The selection of NMP (N-Methyl-2-pyrrolidone) as the solvent for our PVDF–Nafion blend membranes primarily stemmed from its optimal solvency and volatility, which are vital for ensuring uniform and homogeneous membrane casting. Additionally, the literature suggests a potential for incidental porosity enhancement in PVDF membranes when using NMP, although this aspect was not the primary focus of our solvent choice [12]. The blend ratios of PVDF to Nafion were maintained at 90:10, 80:20, 70:30, 60:40, and 50:50. The solution was stirred for over 12 h to ensure homogeneity. The resultant mixture was blade-coated onto a slide glass using a 10 µm spacer and dried at 70 °C. This was followed by annealing at 140 °C to finalize the membrane’s structure.

### 2.2. Stress–Strain Curve Testing

To accurately evaluate the mechanical properties of the materials used in our study, samples for stress–strain curve testing were specially prepared. This approach ensured that the mechanical strength assessment was conducted independently of any specific membrane shape or application form, focusing purely on material characteristics. The preparation of the samples for this testing adhered to the protocols outlined in Section 2.1. Spacers of precisely 125 µm were employed to ensure the uniform thickness of the film during casting. This preparation step was crucial for maintaining consistency across all samples, thereby ensuring reliable mechanical testing outcomes. The films underwent drying and annealing processes similar to those described for membrane synthesis, ensuring the samples’ material properties were consistent for testing. Mechanical properties were then assessed using a universal testing machine at a test speed of 20 mm/min. During the tests, both strain and the standard force exerted on each sample composition were measured.

### 2.3. Electrochemical Measurements

For both selectivity and I–V curve measurements, the membranes were affixed to a 6 mm diameter hole in adhesive PET and positioned in the center of a measurement cell. Ag/AgCl plate electrodes were placed at both ends of the cell, and an Ag/AgCl Luggin capillary electrode was close to the membrane’s surface. For selectivity measurements, the cells were filled with a 10 mM KCl solution on one side and a 500 mM KCl solution on the other. The voltage was recorded using a potentiostat, from which ion transport numbers were calculated. I–V characteristics were determined under varying concentrations. The cells were filled with KCl solutions ranging from 1 to 1000 mM, and the potential was swept from 0 to 1.5 V to generate I–V curves. For concentration gradient-driven conversion efficiency, the setup is as follows: sweeps were conducted from 0.1 V to 0.01 V and 10 mM to 500 mM KCl solutions.

## 3. Results and Discussion

### 3.1. Mechanical Strength Characterization

**Figure 1:**
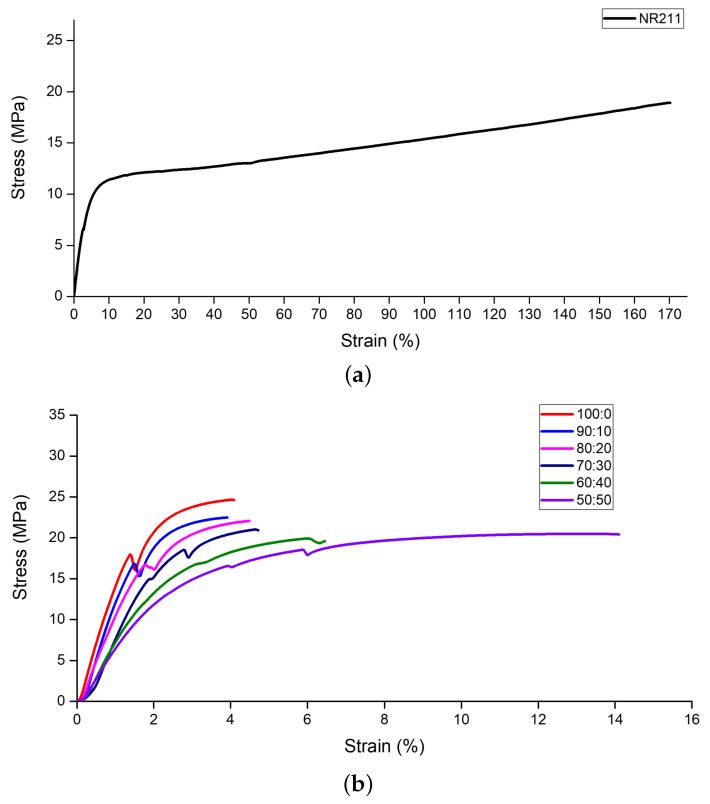
Stress–strain curves of IEMs: (**a**) commercial Nafion NR211 membrane, (**b**) blended composition samples with varying ratios of PVDF to Nafion.

In this section, we present the mechanical strength characterization of our ion exchange membranes (IEMs) with varying compositions of polyvinylidene fluoride (PVDF) to Nafion blends. The mechanical properties were assessed through stress–strain curve testing to ascertain the variations in strength due to different blending ratios, as showcased in Figure 1.

It was hypothesized that PVDF would exhibit superior mechanical properties compared to Nafion, owing to its inherent material characteristics. Benchmarking against commercial Nafion NR211 with a 100:0 ratio of PVDF:Nafion, we observed that Nafion NR211 exhibited an elongation at break of 170.19% and a tensile strength of 18.92 MPa.

In contrast, pure PVDF demonstrated an elongation at break of 4.12% and a higher tensile strength of 24.67 MPa, corroborating our initial assumption. The modulus of elasticity for PVDF was also significantly higher, approximately four times that of NR211, suggesting that Nafion deforms more readily under lower forces compared to PVDF.

The implications of these findings suggest that a substantial PVDF content may be necessary to fabricate thin membranes without compromising mechanical integrity. As the Nafion content was increased up to a 50:50 PVDF:Nafion ratio, we noted a trend:tensile strength and modulus of elasticity decreased, while the strain at break increased. This indicates a trade-off between membrane flexibility and strength as Nafion content rises, an important factor in the design of IEMs where both durability and flexibility are required.

### 3.2. Membrane Thickness Characteristics

**Figure 2:**
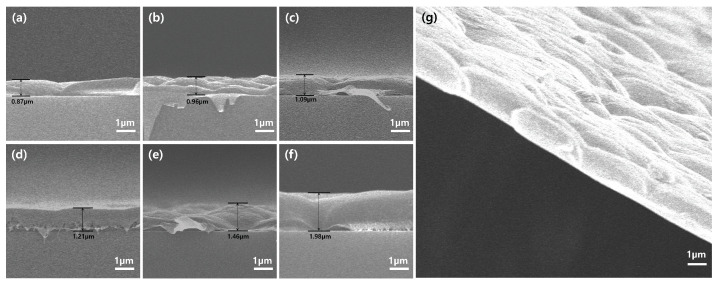
Cross-sectional images through scanning electron microscopy (SEM) revealing the morphology of PVDF:Nafion blend membranes with varying ratios: (**a**) 100:0, (**b**) 90:10, (**c**) 80:20, (**d**) 70:30, (**e**) 60:40, and (**f**) 50:50. (**g**) Angled view of the 50:50 blend, offering insight into the three-dimensional membrane architecture.

The SEM images (Figure 2) not only provide a clear indication of the varying thicknesses of IEMs composed of different PVDF to Nafion ratios but also confirm the successful fabrication of ultra-thin membranes. Remarkably, despite the reduced thicknesses, ranging from 0.87 µm in the pure PVDF membrane (Figure 2a) to 1.98 µm in the 50:50 PVDF:Nafion blend (Figure 2f), all the membranes exhibited freestanding capabilities and could be handled effectively for experimental purposes. This highlights the practical feasibility of employing such thin films in applications, addressing potential concerns about their mechanical stability and handling.

As the proportion of Nafion increases, a corresponding increase in the thickness of the membranes is observed. This variation suggests that the material composition, especially the addition of Nafion, has a direct impact on the membrane’s physical dimensions. The increase in thickness with higher Nafion content might be attributed to Nafion’s higher molecular weight and the resulting viscosity, which could result in a denser film during the blade-coating process [13]. Additionally, the ionic nature of Nafion may affect the solvent’s evaporation rate, leading to a thicker membrane after drying [14]. While not the primary focus of this analysis, it is noteworthy that the membrane surfaces across all ratios are not entirely smooth. The subtle variations in surface texture, however, do not seem to compromise the overall uniformity and integrity of the membranes.

Having primarily focused on the physical properties of the membranes in the analysis, another critical aspect to be discussed in the subsequent sections is the retention of ion exchange characteristics in these blends’ content. As the functional performance of the membranes, particularly in terms of ion transport and resistance, hinges on the presence and effectiveness of ion-exchange groups [15], it is crucial to verify whether these membranes maintain the ion exchange properties that are fundamental to Nafion’s efficacy in applications like salinity gradient energy harvesting [16,17]. Our upcoming results provide a comprehensive understanding of how blend composition influences both the structural and operational aspects of IEMs.

### 3.3. Permselectivity in Relation to IEC

In exploring the functionality of IEMs with varying Nafion contents, our investigation delves into the nuanced relationship between ion exchange capacity (IEC) and permselectivity. Contrary to the traditional belief that a higher IEC is indispensable for optimal permselectivity [18,19,20], our findings, aligned with recent studies, demonstrate that membranes with lower Nafion content can also exhibit high selectivity [21]. This observation suggests that, particularly for our PVDF–Nafion blend membranes, excellent permselectivity may be achieved without the necessity of high IEC levels. The examination of the IEMs’ functionality commenced with the calculation of the IEC for each blend, as shown in Table 1. These IEC values were derived employing the equation IEC = 1000/EW, where EW, equivalent weight, was calculated from the Nafion ratio in each blend [22].

To further understand permselectivity, we measured the membrane potential ($\Delta E_{m}$) under a concentration gradient and calculated the ion transport number ($t_{+}$) using the following equation [23]:

(1) $$\Delta E_{m}=\left(2t_{+}-1\right)\frac{RT}{zF}\ln\frac{c_{h}}{c_{l}}$$

where $\Delta E_{m}$ represents the membrane potential, $t_{+}$ is the transference number of the cation, *R* is the gas constant, *T* is the temperature in Kelvin, *z* is the charge number of the ion, *F* is Faraday’s constant. Here, $c_{h}$ represents the ion concentration on the side of the membrane facing the higher-concentration solution, and $c_{l}$ represents the ion concentration on the side facing the lower-concentration solution.

**Figure 3:**
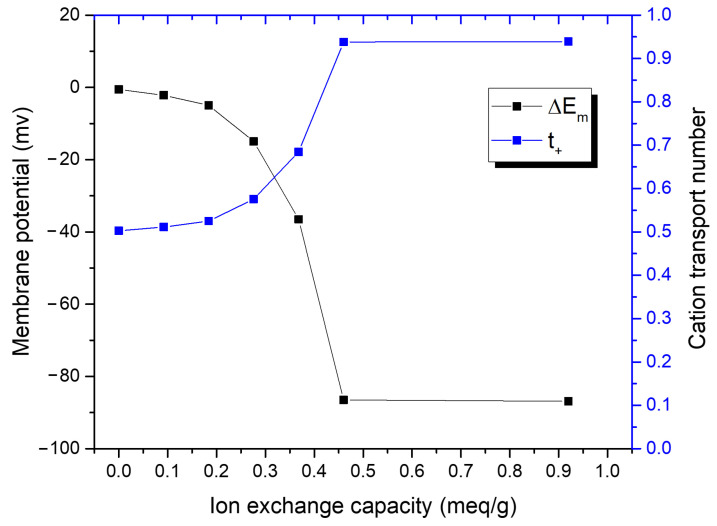
Correlation of IEC with membrane potential and cation transport number across varying Nafion contents.

These results are graphically represented in Figure 3, where a clear trend emerges: membranes with higher Nafion content exhibited increased permselectivity. Astonishingly, the 50:50 PVDF:Nafion blend demonstrated permselectivity on par with commercial Nafion membranes, despite its IEC being only half as much. This finding suggests that there is a threshold in Nafion content beyond which additional IEC does not translate to proportionately higher permselectivity. This has significant understanding for the design of IEMs, indicating that optimal performance can be achieved with an approach that adjusts material composition.

This finding extends the practical application of IEMs in salinity gradient energy harvesting. While a certain level of IEC is undoubtedly necessary for efficient ion exchange, our findings suggest that, beyond a certain point, the benefits of increased Nafion content taper off. Thus, the 50:50 blend represents an optimal trade-off between material efficiency and functional performance. In conclusion, the structural and functional analyses of our membrane blends reveal a complex interaction between material composition and membrane functionality. These could lead to the development of next-generation IEMs that are both cost-effective and high performance, potentially broadening the scope of their application in the field of electrochemistry.

### 3.4. Concentration-Dependent I–V Curves

**Figure 4:**
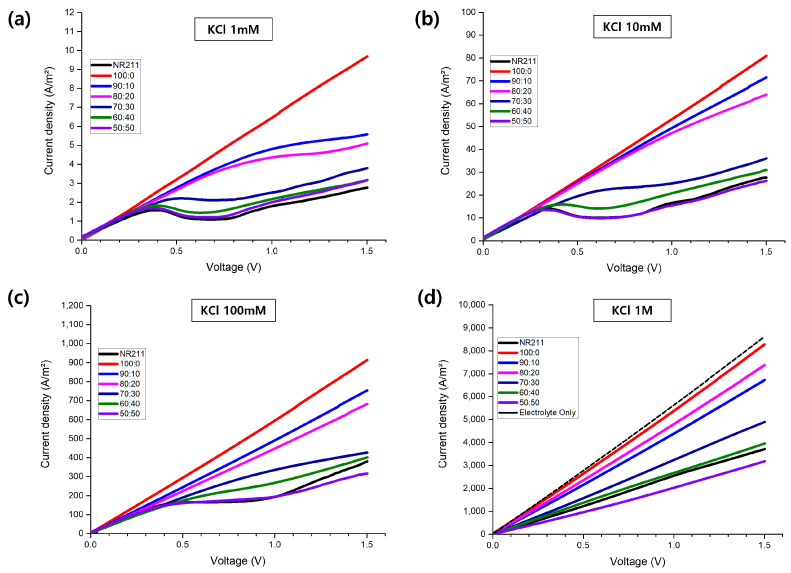
Current density–voltage characteristics of PVDF–Nafion blend membranes, illustrating the membranes’ performance in different electrolytic environments: (**a**) 1 mM KCl, (**b**) 10 mM KCl, (**c**) 100 mM KCl, and (**d**) 1 M KCl.

In the section, we investigate the current density–voltage behavior of our membrane samples at varying concentrations. As evidenced in Figure 4, the pure PVDF membrane (100:0 ratio) displays notable ionic conductivity, contrary to the conventional understanding that PVDF, being inherently non-ionic, would not support effective ion transport. This indicates that interconnected free volumes within the thin PVDF structure might facilitate ion movement, suggesting a potential for non-ion-selective ion transport [24]. With increasing Nafion content, a shift towards more selective ion transport was observed, underscoring Nafion’s role in enhancing membrane selectivity. From pure PVDF to membranes with more Nafion, the following two trends become apparent. First, a shift in the voltage for limiting current density appears across concentrations from 1 mM to 100 mM, and higher Nafion content leads to earlier limiting current density onset at lower voltages due to enhanced ion-selective properties. This limiting current density reflects concentration polarization effects and is aligned with the membrane’s selectivity, as shown in Figure 3 [25,26].

Even with a considerable PVDF content, the 50:50 PVDF:Nafion blend achieves high selectivity. This indicates that Nafion effectively harnesses PVDF’s free volume, thus minimizing non-selective pathways. The relationship between increased Nafion content and conductivity can also be discerned by examining the linear fitting data from the 1 M KCl curve. These data, which have been used to calculate membrane resistance and conductivity, are presented in Table 2, elucidating the impact of Nafion on reducing non-selective pathways. The conductance per unit area ($\frac{dI}{dV}\cdot \frac{1}{A}$, measured in S/m^2^), obtained from the linear fit of the I–V curve, is pivotal in evaluating the ionic transport properties of the membranes. By taking the reciprocal of this value, we can determine the combined resistance of the solution ($R_{s}$) and the membrane ($R_{m}$), expressed as $(R_{s}+R_{m})\cdot A$ [27]. This gives us a comprehensive measure of the resistance to ionic transport across the membrane system. The intrinsic resistance of the membrane ($R_{m}$, in Ωm^2^) is obtained by subtracting the solution resistance from the combined resistance, thereby providing a quantitative assessment of the membrane’s opposition to ion movement [28]. The $\sigma_{\mathrm{slope}}$ value reflects the accuracy of the slope determination, and the $R^{2}$ value gives us the degree of fit of the regression line to the data points.

**Figure 5:**
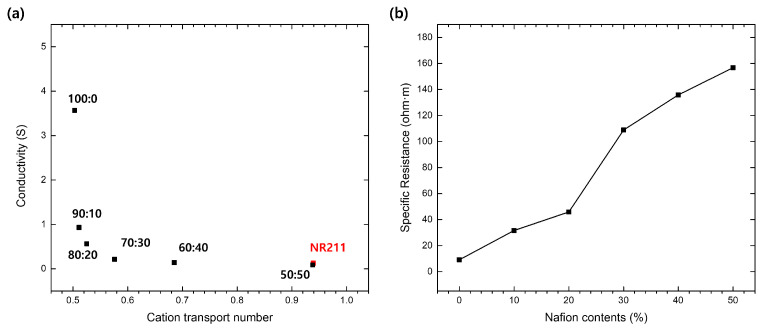
Electrochemical characterization of IEMs with varying ratios of PVDF to Nafion, showing the interplay between ionic selectivity and conductivity: (**a**) the relationship between membrane conductivity and cation transport number across different blend ratios; (**b**) the specific membrane resistance as a function of Nafion content.

Upon examining the solution resistance, we determined the overall resistance and conductivity of our membranes, as depicted in Figure 5a. This analysis revealed a nuanced interplay between conductivity and selectivity, underlining the complexity inherent in optimizing membrane performance. Acknowledging the well-established principle that an increased Nafion or ionomer content generally boosts conductivity, our observations of conductivity reduction pertain specifically to the distinct characteristics of our PVDF—Nafion blend membranes. Given this context, we anticipate that, under typical conditions, an uptick in Nafion content would substantially enhance conductivity through improved selective ion transport over non-selective pathways. Such an enhancement in selective ion transport with greater Nafion incorporation corroborates the observed uplift in permselectivity across our blend membranes, affirming the positive influence of Nafion content on both conductivity and selectivity.

Nevertheless, isolating the impacts of Nafion content from membrane thickness on performance necessitates further investigation [29]. Therefore, an in-depth analysis is essential to unraveling the specific contributors to this observed conductivity variation. Figure 5b, with adjustments for membrane thickness, demonstrates a rise in resistance accompanying increased Nafion content. This pattern suggests that the conductivity reduction observed is not merely a consequence of augmented membrane thickness but also results from alterations in the membrane’s internal composition by Nafion.

This adjustment in conductivity is chiefly ascribed to a decrease in PVDF content and the associated reduction in non-selective transport pathways, rather than an increase in Nafion content. By constraining PVDF’s non-selective free volumes, Nafion introduces a more regulated and selective ion transport mechanism, naturally exhibiting higher resistance compared to the broader pathways present in PVDF alone. This strategic increase in resistance, aiming to boost selectivity and effectively manage ion transport, is pivotal for the membranes’ efficacy in energy harvesting applications.

The resulting increase in resistance is a deliberate balance, aimed at enhancing selectivity and managing ion transport more effectively. This balance is crucial for membrane functionality in energy harvesting applications, where both high selectivity and efficient conductivity are essential.

### 3.5. Energy Harvesting Performance

**Figure 6:**
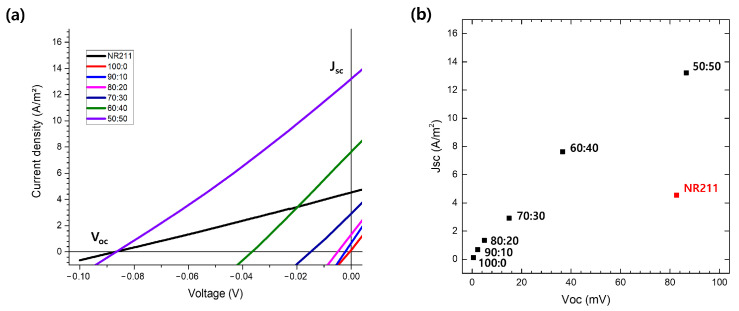
Electrochemical performance of IEMs with varying PVDF to Nafion ratios: (**a**) current density–voltage curves demonstrating the open-circuit voltage (V_oc_) and short-circuit current density (J_sc_) for different membrane compositions; (**b**) correlation between V_oc_ and J_sc_ across the membranes, with the 50:50 PVDF:Nafion blend exhibiting higher J_sc_ at comparable V_oc_ levels to the commercial NR211 membrane.

The energy harvesting capabilities of our IEMs are compellingly demonstrated in Figure 6a, where the open-circuit voltage (V_oc_) and the short-circuit current density (J_sc_) are measured. V_oc_ represents the maximum potential difference the membrane can develop without an external load, while J_sc_ reflects the maximum current density achievable when the circuit is closed without external resistance. These parameters are pivotal in ascertaining the potential power density of a membrane, a key metric for energy harvesting applications [30]. In Figure 6b, the relationship between V_oc_ and J_sc_ is graphically represented, extracted from the intersection points of the curves in Figure 6a with the axes. Theoretically, the power density (P) of an energy harvesting system can be calculated as P = V_oc_ × J_sc_. The maximum power density (P_max_), representing the optimal operation point, is typically at half the values of V_oc_ and J_sc_, indicating P_max_ = (V_oc_/2) × (J_sc_/2) [31]. A notable observation from Figure 6b is that the 50:50 PVDF:Nafion blend exhibits a J_sc_ superior to that of the commercial Nafion membrane NR211 at comparable V_oc_ levels, suggesting the potential for higher power density under analogous operating conditions.

This enhanced J_sc_, particularly under a salinity gradient, implies that factors beyond mere conductivity are influencing ion transport. Despite observing lower conductivity in 1M KCl tests, the blended membrane facilitates ion diffusion more efficiently under gradient conditions. Ion diffusion, a key factor in the heightened J_sc_ under a salinity gradient, reveals that the blending of membranes offers unique attributes conducive to ion transport [32]. The factors promoting ion diffusion might be partly attributed to the surface characteristics observed in SEM images. For instance, the uneven, rippled surface topography seen in Figure 2g could enhance ion diffusion. This microstructural complexity may increase the effective surface area for ion transport, contributing to enhanced current generation under a salinity gradient [33]. Moreover, the heterogeneity of the membrane’s surface likely creates localized regions where diffusion is more pronounced, resulting in steeper concentration gradients driving the diffusion process [34]. The blend’s tailored microstructure and optimized surface characteristics contribute to effective ion diffusion.

## 4. Conclusions

Ultra-thin membranes were successfully fabricated with varying PVDF to Nafion ratios and maintained mechanical durability despite reduced thickness. Membranes with lower Nafion content (as low as 50%) not only achieved permselectivity comparable to higher Nafion content membranes but also exhibited higher energy harvesting performance than Nafion at a 50:50 ratio of PVDF to Nafion. These are due to the blend’s tailored microstructure and optimized surface characteristics, which effectively enhance ion diffusion. These findings will not only contribute to the fundamental understanding of membrane science but also open new avenues for the application of IEMs in the field of electrochemistry.

## Figures and Tables

**Table 1:** Theoretical equivalent weight (EW) and ion exchange capacity (IEC) based on the Nafion ratio in the blend.

Sample	Nafion Content (wt%)	EW * (g/eq)	IEC (meq/g)
NR211, D2021	100	1100	0.910
100:0	-	-	-
90:10	10	110	0.091
80:20	20	220	0.182
70:30	30	330	0.273
60:40	40	440	0.364
50:50	50	550	0.455

* EW calculated as 1100 times the Nafion content.

**Table 2:** Data for extracting conductance and resistance.

Sample	$\frac{\mathrm{dI}}{\mathrm{dV}}\cdot \frac{1}{A}$ (S/m^2^)	$(R_{s}+R_{m})\cdot A$, $R_{m}\cdot A$ (Ωm^2^)	$\sigma_{\mathrm{slope}}$, $R^{2}$
Electrolyte	5669.48	$1.76\times 10^{-4}$, -	4.36347, 0.99990
NR211	2508.16	$3.99\times 10^{-4}$, $2.22\times 10^{-4}$	2.42269, 0.99986
100:0	5425.63	$1.84\times 10^{-4}$, $7.93\times 10^{-6}$	5.47738, 0.99985
90:10	4836.86	$2.07\times 10^{-4}$, $3.04\times 10^{-5}$	4.60411, 0.99986
80:20	4417.23	$2.26\times 10^{-4}$, $5.00\times 10^{-5}$	3.94884, 0.99988
70:30	3245.42	$3.08\times 10^{-4}$, $1.32\times 10^{-4}$	2.73311, 0.99989
60:40	2668.45	$3.75\times 10^{-4}$, $1.98\times 10^{-4}$	3.18344, 0.99979
50:50	2054.14	$4.87\times 10^{-4}$, $3.10\times 10^{-4}$	4.66627, 0.99923

## Data Availability

Data are contained within the article.
